# Factors Associated With Limited Digital Health Literacy Among Chinese Male Populations: Cross-sectional Study

**DOI:** 10.2196/42868

**Published:** 2023-04-19

**Authors:** Zhaoquan Xing, Meng Ji, Zhaogang Dong, Xiaofei Xu, Yi Shan

**Affiliations:** 1 Department of Urology Qilu Hospital of Shandong University Ji'nan China; 2 School of Languages and Cultures The University of Sydney Sydney Australia; 3 Department of Clinical Laboratory Qilu Hospital of Shandong University Ji'nan China; 4 Center for Reproductive Medicine Department of Obstetrics and Gynecology Qilu Hospital of Shandong University Ji'nan China; 5 School of Foreign Studies Nantong University Nantong China

**Keywords:** factor, older age, lower education attainment, lower functional, communicative, and critical health literacy, weaker belief and self-confidence, limited eHealth literacy, Chinese population, logistic regression

## Abstract

**Background:**

eHealth resources and interventions promise to promote favorable behavior change, self-efficacy, and knowledge acquisition, thereby improving health literacy. However, individuals with limited eHealth literacy may find it difficult to identify, understand, and benefit from eHealth use. It is necessary to identify the self-assessed eHealth literacy of those who use eHealth resources to classify their eHealth literacy levels and to determine the demographic characteristics associated with higher and lower eHealth literacy skills.

**Objective:**

This study aimed to identify notable factors closely associated with limited eHealth literacy among Chinese male populations to provide some implications for clinical practice, health education, medical research, and public health policy making.

**Methods:**

We hypothesized that participants’ eHealth literacy status was associated with various demographic characteristics. Therefore, we elicited the following information in the questionnaire: age and education, self-assessed disease knowledge, 3 well-developed health literacy assessment tools (ie, the All Aspects of Health Literacy Scale, eHealth Literacy Scale, and General Health Numeracy Test), and the 6 *Internal* items on health beliefs and self-confidence in the Multidimensional Health Locus of Control Scales. Using randomized sampling, we recruited survey participants from Qilu Hospital of Shandong University, China. After validating the data collected through a web-based questionnaire survey via *wenjuanxing*, we coded all valid data according to predefined coding schemes of Likert scales with different point (score) ranges. We then calculated the total scores of the subsections of the scales or the entire scale. Finally, we used logistic regression modeling to associate the scores of the eHealth Literacy Scale with the scores of the All Aspects of Health Literacy Scale, the General Health Numeracy Test-6, and age and education to ascertain factors considerably associated with limited eHealth literacy among Chinese male populations.

**Results:**

All data from the 543 returned questionnaires were valid according to the validation criteria. By interpreting these descriptive statistics, we found that 4 factors were significantly correlated with participants’ limited eHealth literacy: older age, lower education attainment, lower levels of all aspects of health literacy (functional, communicative, and critical), and weaker beliefs and self-confidence in internal drivers and strengths to stay healthy.

**Conclusions:**

By applying logistic regression modeling, we ascertained 4 factors that were significantly correlated with limited eHealth literacy among Chinese male populations. These relevant factors identified can inform stakeholders engaging in clinical practice, health education, medical research, and health policy making.

## Introduction

### Background

Literacy related to health information is becoming a critical factor relevant to health status [1,2]. Patients with low health literacy are likely to lack the skills essential for effectively interacting with the health system and engaging in appropriate self-care, including the know-how to take medications and interpret labels and other health information [3]. Previous studies have linked limited health literacy to poorer health status, increased hospitalization, nonadherence to medications, medication dosing errors, and increased mortality [1,4-6]. Therefore, it is necessary to deliver effective interventions to address low health literacy. Existing interventions rely mainly on communication and education alone; therefore, they mostly fail to achieve substantive, sustained behavioral changes [7]. One potential solution to low health literacy is to develop cost-effective interventions that can be easily understood, acceptable, deployed, and readily accessible on the web [3]. The importance of such eHealth interventions has been well-documented in the literature. For example, Eland-de Kok et al [8] found that eHealth interventions for chronic diseases could have positive effects on health outcomes. Santana et al [9] discovered that almost 27% of European people who had sought health information on the internet actively suggested diagnoses or treatments to their physician and thus played more active roles in making medical decisions [9]. Or and Tao [10] revealed that the use of eHealth technologies positively influenced clinical parameters, including blood pressure or cholesterol levels.

The effective delivery and uptake of eHealth interventions calls for eHealth knowledge and skills necessary to navigate health-related websites, platforms, and systems, that is, eHealth literacy. eHealth literacy has been defined as “the ability to seek, find, understand, and appraise health information from electronic sources and apply the knowledge gained to addressing or solving a health problem” by Norman and Skinner [11] to address the emerging need to understand users’ digital competencies in the health context. They developed the 8-item eHealth Literacy Scale (eHEALS) to assess eHealth literacy [12]. According to them, a user’s eHealth literacy comprises 3 types of literacy (ie, health, computer, and science literacy) and 3 types of analytical literacy (ie, traditional, information, and media literacy) [11]. Chan et al [13] expanded this model to include additional taxonomic levels for the 6 subsets of literacy above, and Gilstad [14] added contextual, cultural, and social dimensions. On the basis of the European Health Literacy Survey [15], Bautista [16] defined eHealth literacy anew as “the interplay of individual and social factors in the use of digital technologies to search, acquire, comprehend, appraise, communicate and apply health information in all contexts of health care with the goal of maintaining or improving the quality of life throughout the lifespan.” eHealth literacy plays an essential role in maintaining and promoting health [17]. Individuals with high eHealth literacy not only tend to use the internet to find answers to health-related issues but also better understand the information sought on the web, verify its veracity, and use it to promote health behaviors [18].

Personal characteristics and experiences unique to each individual can impact their subsequent health-promoting behaviors [19]. Those with better health literacy are most likely to be skillful and capable of engaging in various health-enhancing actions [20]. eHealth resources and interventions have promised to promote favorable behavior change, self-efficacy, and knowledge acquisition, thus improving health literacy [21]. However, individuals with limited eHealth literacy may find it difficult to identify, understand, and benefit from their use [22]. Although eHealth literate people are likely to be motivated and able to find, appraise, integrate, and apply eHealth resources [23], only those who can adequately combine literacy and skills can derive actual health benefits [24]. In this context, it is necessary to identify the self-assessed eHealth literacy of those who use eHealth resources, to classify their eHealth literacy levels, and to determine what demographic characteristics may be associated with higher and lower eHealth literacy skills. As a result, we can inform relevant stakeholders, including health care and medical providers, health educators, health and medical researchers, and public policy makers, who could thus implement more targeted eHealth-related interventions across various diseases. However, no studies have been conducted to investigate the contributing factors or predictors of limited and adequate eHealth literacy skills among Chinese people.

There are mixed findings regarding the association between sex and health literacy. Some studies have ascertained an association between male sex and limited health literacy [25-29]. However, other studies have found that poor health literacy is closely related to female sex [30,31]. The mixed findings on the association between sex and health literacy can add important information to the growing understanding of the role of sex in health literacy [27]. These inconsistent findings warrant future studies conducted in different linguistic, cultural, ethnic, and socioeconomic communities to further pinpoint the association between sex and health literacy levels [29]. As a very recent study has revealed a correlation between male sex and low critical health literacy (CRHL) among the Chinese population [29], we intended to determine whether digital health literacy could be closely correlated with male sex among Chinese populations.

### Objective

This study aimed to identify notable factors closely associated with limited eHealth literacy among Chinese male populations to provide some implications for clinical practice, health education, medical research, and public health policy making.

## Methods

### Questionnaire Design

The questionnaire included the following information: (1) age and education, (2) self-assessed disease knowledge, (3) 3 well-developed health literacy assessment tools (ie, the All Aspects of Health Literacy Scale [AAHLS] [32], eHEALS [33], and General Health Numeracy Test [GHNT-6] [34]), and (4) the 6 *Internal* items on health beliefs and self-confidence in the Multidimensional Health Locus of Control (MHLC) scales [35].

Previous studies have found a strong association between poor eHealth literacy and lower levels of functional, communicative, and CRHL [36-40].

The “Internal” locus of control has been found to be associated with health-promoting behaviors, health risk-reducing actions, and knowledge about health problems [41-45]. On the basis of previous studies, we hypothesized that participants’ eHealth literacy status could be associated with factors such as age, education, self-assessed disease knowledge, carious health literacy skills, and the “Internal” locus of control.

### Informant Recruitment and Web-Based Survey

Using randomized sampling, we recruited survey participants from the Qilu Hospital of Shandong University, China. Those included in this study must (1) be aged ≥18 years, (2) have 6 years of or over of schooling experience to understand the questionnaire item, and (3) voluntarily participate in the survey. We conducted face-to-face contact with Chinese patients attending the outpatient clinic of Qilu Hospital and those who were hospitalized in this hospital to identify those who satisfied the inclusion criteria, informed them about the purpose of the survey, and asked them to participate in the web-based survey as scheduled. We conducted a power analysis to determine a sample size of 218 participants. A total of 589 eligible patients were included.

The questionnaire was administered via *wenjuanxing* [46], the most popular digital survey platform in China, from July 26 to August 25, 2022. The returned questionnaire was valid only when all the question items included were answered according to our predefined validation criterion.

### Data Collection, Coding, and Analysis

On August 26, 2022, responses to the questionnaire were downloaded from *wenjuanxing* and stored in a Microsoft Excel file. A total of 543 questionnaires were returned, with a response rate of 92.2% (543/589). We double-checked whether a response was returned for each question item to ascertain the validity of the data in each returned questionnaire. Subsequently, all valid data were coded according to predefined coding schemes of Likert scales with different point (score) ranges. We then calculated the total scores of the subsections of the AAHLS and the total scores of the 2 health literacy scales (eHEALS and GHNT-6). Finally, we used logistic regression modeling to associate the scores of the eHEALS with the scores of the AAHLS, the GHNT-6, and age and education to ascertain factors significantly associated with limited eHealth literacy among Chinese male populations.

### Ethics Approval

This study was approved by the Ethics Review Board of the Qilu Hospital of Shandong University, China (KYLL-202208-026). The study data were anonymized to protect the privacy and confidentiality of the study participants. As the participants voluntarily participated in the survey to support and promote academic research, no compensation was provided for them as per the common practice in China.

## Results

### Descriptive Statistics of the Information Collected

Multimedia Appendix 1 presents descriptive statistics of the data collected from the informants. All data from the 543 returned questionnaires were valid according to the validation criteria. The informants were aged 45.73 (SD 10.31) years on average. All of them were male. The mean score for education was 2.91 (SD 1.35), indicating that the informants’ average educational attainment was just below year 12 of schooling. The mean score for their self-assessed disease knowledge (2.34, SD 0.98) indicates that they rated their disease knowledge between “knowing a lot” and “knowing some.” The mean scores of the subconstructs in the AAHLS were 6.83 (SD 1.57) for functional health literacy (FHL), 5.73 (SD 1.45) for communicative health literacy (COHL), and 11.33 (SD 2.009) for CRHL. These mean values imply that they *sometimes* needed help to read health-related information, they *sometimes* knew how to effectively communicate with doctors and nurses, and they were *sometimes* critical about health information, respectively. The mean score for each question in the GHNT was determined as 1.55 (SD 0.50), 1.12 (SD 0.33), 1.14 (SD 0.35), 1.94 (SD 0.27), 1.88 (SD 0.32), and 1.84 (SD 0.37), respectively. Each participant returned an average of 2.52 (SD 1.00) correct responses to the 6 numeracy questions. These mean scores show that a large proportion of participants answered the 6 questions in the GHNT incorrectly, especially questions 1, 4, 5, and 6. The mean score for eHealth literacy was 22.01 (SD 4.50), indicating that they were unsure about their ability to use eHealth resources and interventions.

The distribution of eHealth literacy sum scores among male participants shows that most participants scored between 16 and 26. This score range means that most participant returned a “disagree” or “unsure” response to the 8 questions items in the eHEALS. In other words, they did not think that they could effectively use eHealth resources or they were unsure about their eHealth literacy skills.

Drawing on Turkey hinges, we identified 3 thresholds of eHealth literacy among Chinese male participants (valid number: 543): inadequate=13-22 (representing 50% of the sum score for the Chinese version of the eHEALS [CH-eHEALS]), problematic=23-24 (representing 25% of the sum score for CH-eHEALS), and sufficient=25-40 (representing 25% of the total score for CH-eHEALS), as shown in Table 1. This threshold resolution was different from that determined by Wångdahl et al [47]: inadequate=8-20 (representing 50% of the sum score for the Swedish version of the eHEALS [Sw-eHEALS]), problematic=21-26 (representing 25% of the sum score for Sw-eHEALS), and sufficient=27-40 (representing 25% of the sum score for Sw-eHEALS) [47]. This difference in the determination of thresholds suggests that threshold levels for the eHEALS should be further evaluated in different populations and languages [47]. Drawing on the 3 thresholds identified, we ascertained the proportions of the 3 literacy clusters: inadequate (311/543, 57.3%), problematic (100/543, 18.4%), and sufficient (132/543, 24.3%). We combined the 2 groups of inadequate and problematic eHealth literacy into 1 category of limited eHealth literacy and then used logistic regression to explore factors associated with limited eHealth literacy among Chinese male populations.

**Table 1:** Thresholds of eHealth literacy scale (eHEALS): inadequate (22 and below), problematic (23-24), and sufficient (25 or above).

	Percentiles						
	5	10	25	50	75	90	95
Weighted average, eHEALS_SUM^a^	16.00	17.00	19.00	22.00	24.00	28.00	31.00
Tukey hinges, eHEALS_SUM	N/A^b^	N/A	19.00	22.00	24.00	N/A	N/A

^a^eHEALS_SUM: the sum scores of the eHEALS.

^b^N/A: not applicable.

### Multilinearity Statistics of the Predictor Variables

#### Multicollinearity

The collinearity statistics, including the variance inflation factor and tolerance in Table 2, show that the correlation among the 8 predictor variables was at an acceptable level, as all variance inflation factor scores were lower than 2 and their matching tolerance scores were smaller than 1 [47,48]. The 8 predictor variables encompass age, educational attainment, self-reported disease knowledge, sum of FHL [32], sum of COHL [32], sum of CRHL [32], sum of correct responses to the 6 questions in the GHNT-6 [34], and sum of the 6 “Internal” items on health beliefs and self-confidence in the MHLC scales [35].

**Table 2:** Collinearity statistics.

Predictor variables	Tolerance	VIF^a^
Age	0.93	1.08
Education	0.85	1.17
Disease knowledge	0.99	1.01
FHL_SUM^b^	0.94	1.06
COHL_SUM^c^	0.87	1.16
MHLC_SUM^d^	0.86	1.17
GHNT_SUM^e^ of correct responses	0.99	1.01
CRHL_SUM^f^	0.97	1.03

^a^VIF: variance inflation factor.

^b^FHL_SUM: sum of the functional health literacy subscale of the All Aspects of Health Literacy Scale.

^c^COHL_SUM: sum of the communicative health literacy subscale of the All Aspects of Health Literacy Scale.

^d^MHLC_SUM: sum of the Multidimensional Health Locus of Control scales.

^e^GHNT_SUM: sum of the General Health Numeracy Test responses.

^f^CRHL_SUM: sum of the critical health literacy subscale of the All Aspects of Health Literacy Scale.

Table 3 shows that age and education levels were important predictors of limited eHealth literacy. Specifically, with an increase of 1 year in age, the odds of male participants with limited eHealth literacy increased by 2% (age: odds ratio [OR] 1.02, 95% CI 1.00-1.05; *P*=.03). In the regression modeling process, the reference category for education was postgraduate or above. The results showed that when a male participant had completed the lowest level of education (year 6), the odds of being in the limited eHealth literacy group increased by 2369% (year 6: OR 24.69, 95% CI 2.71-224.85; *P*<.001). When a male participant had completed junior high school (year 9), senior high school (year 12), 3-year college diploma, or a 4-year first degree at a Chinese university, the odds of being in the limited eHealth literacy group increased by 1575% (year 9: OR 16.75, 95% CI 1.90-147.38; *P*=.01), 1601% (year 12: OR 17.01, 95% CI 1.93-149.92; *P*=.01), 1472% (diploma: OR 15.72, 95% CI 1.79-137.68; *P*=.01), and 1979% (university: OR 20.79, 95% CI 2.32-186.53; *P*=.01), respectively.

Limited eHealth literacy was also strongly associated with a range of health knowledge, skills, behaviors, and beliefs as measured by the FHL and COHL subscales of the AAHLS and the MHLC. The second item of the FHL subscale was, “When you need help, can you easily get hold of someone to assist you?” The responses were coded as 1=not applicable, 2=rarely, 3=sometimes, and 4=often, and “often” was used as the reference category. It was found that when a Chinese male participant was “rarely” able to easily secure help from others when needing help, the odds of being in the limited eHealth literacy category increased by 232% (FHL2, rarely: OR 3.32, 95% CI 1.62-6.82; *P*<.001).

The second item (COHL2) of the COHL was “When you talk to a doctor or nurse, do you ask the questions you need to ask?” We coded the responses in the same way as for the FHL subscale. It was found that compared with male participants who “rarely” asked the questions that they needed to ask health professionals, among those who responded “often” and “sometimes,” their odds of being in the limited eHealth literacy group decreased significantly by 64% (COHL2, often: OR 0.36, 95% CI 0.19-0.69; *P*<.001) and 55% (COHL2, sometimes: OR 0.45, 95% CI 0.24-0.84; *P*=.01).

Finally, we used the MHLC (Form A) to measure health beliefs among male participants. Responses were coded as 1=strongly disagree, 2=moderately disagree, 3=slightly disagree, 4=slightly agree, 5=moderately agree, and 6=strongly agree. Thus, an increase in the sum scores of MHLC questions indicated strong internal drivers in managing one’s own health. The results showed that with an increase of one unit in the sum score of the MHLC scales, the odds of a Chinese male participant being in the limited eHealth literacy group decreased by 6% at a statistically significant level (sum of the MHLC scales: OR 0.94, 95% CI 0.90-0.98; *P*<.001).

**Table 3:** Factors associate with limited eHealth literacy among Chinese male populations. Predicted membership is limited eHealth literary.

	B (SE)	Wald (*df*)	*P* value	Exponential, B (95% CI)
Age	0.02 (0.01)	5.01 (1)	.03	1.02 (1.00-1.05)
Education (reference: postgraduate)	N/A^a^	8.96 (5)	.11	N/A
Education (year 6)	3.21 (1.13)	8.09 (1)	<.001	24.69 (2.71-224.85)
Education (year 9)	2.82 (1.11)	6.45 (1)	.01	16.75 (1.90-147.38)
Education (year 12)	2.83 (1.11)	6.52 (1)	.01	17.01 (1.93-149.92)
Education (diploma)	2.75 (1.11)	6.19 (1)	.01	15.72 (1.79-137.68)
Education (university)	3.03 (1.12)	7.35 (1)	.01	20.79 (2.32-186.53)
FHL2^b^ (reference: often)	N/A	12.32 (3)	.01	N/A
FHL2 (not applicable)	0.64 (0.37)	3.04 (1)	.08	1.89 (0.92-3.87)
FHL2 (rarely)	1.20 (0.37)	10.69 (1)	<.001	3.32 (1.62-6.826.82)
FHL2 (sometimes)	0.19 (0.26)	0.52 (1)	.47	1.21 (0.73-2.01)
COHL2^c^ (reference: rarely)	N/A	9.63 (2)	.01	N/A
COHL2 (often)	1.02 (0.33)	9.46 (1)	<.001	0.36 (0.19-0.69)
COHL2 (sometimes)	0.80 (0.32)	6.26 (1)	.01	0.45 (0.24-0.84)
MHLC_SUM^d^	0.06 (0.02)	8.83 (1)	<.001	0.94 (0.90-0.98)
Constant	1.17 (1.30)	0.81 (1)	.37	0.31 (N/A)

^a^N/A: not applicable.

^b^FHL2: item 2 of the functional health literacy subscale.

^c^COHL2: item 2 of the communicative health literacy subscale.

^d^MHLC_SUM: sum of the Multidimensional Health Locus of Control scales.

#### Factors and Health Behaviors Associated With Self-Reported Limited eHealth Literacy by Scale Item

Next, we analyzed factors associated with responses to individual items of eHealth literacy. We labeled the following responses as belonging to the limited eHealth literacy category: “strongly disagree,” “disagree,” and “unsure,” in comparison with the responses of “agree” and “strongly agree.” Informed by Manganello et al [36], we analyzed factors and health behaviors associated with limited eHealth literacy responses using scale items. The first item (eHEALS1) of the eHEALS was “I know what health resources are available on the internet.” Higher sum scores of communicative health literacy indicated lower abilities to engage and interact effectively with health professionals, such as less likely to share information that doctors needed to help the patients (COHL1), less likely to ask health professionals questions that they needed to ask (COHL2), and less likely to ask doctors to explain any information that they did not understand (COHL3). It was found that with an increase of one unit in the sum score of COHL, the odds of a male participant being in the limited eHealth literacy group increased by 25% (sum of the COHL scale: OR 1.25, 95% CI 1.09-1.43; *P*<.001).

We also found that limited eHealth literacy, specifically not knowing “what health resources were available on the internet” (eHEALS1), was strongly associated with an increased tendency and frequency to challenge health and medical professionals based on one’s own research among Chinese male participants. For example, we coded the responses to the fourth item of critical health literacy “Are you the sort of person who might question your doctor or nurse’s advice based on your own research?” as 1=yes, definitely, 2=maybe/sometimes, and 3=not really. The regression modeling used “3=not really” as the reference category and revealed that when a male participant responded “definitely” or “maybe/sometimes,” his odds of being in the limited eHealth literacy group increased significantly by 104% (CRHL4, yes: OR 2.04, 95% CI 1.20-3.46; *P*=.01; and CRHL4, sometimes: OR 2.04, 95% CI 1.28-3.27; *P*<.001), as shown in Table 4.

The second item (eHEALS2) of the eHEALS was “I know where to find helpful health information on the internet.” We found that education continued to be an important predictor of limited eHealth literacy in terms of one’s ability to “find helpful health information on the internet.” Using “postgraduate or above” as the reference educational level, it was found that lower educational levels predicted larger increases in the odds of male participants having trouble identifying helpful health information on the web. The largest increase in the odds of experiencing difficulties in identifying useful health information was found among Chinese male participants with year 6 or below education (OR 10.44, 95% CI 2.78-39.19; *P*<.001), as shown in Table 5. Smaller yet statistically significant increases in the odds of having difficulties in searching for health information were observed among male participants with a year 9 education (OR 5.75, 95% CI 1.63-20.35; *P*=.01), year 12 education (OR 6.23, 95% CI 1.75-22.16; *P*<.001), 3-year college diplomas (OR 6.39, 95% CI 1.79-22.77; *P*<.001), and 4-year university degrees (OR 6.53, 95% CI 1.73-24.67; *P*=.01), as shown in Table 5.

**Table 4:** Factors and health behaviors associated with limited eHealth literacy responses (item 1 of the eHealth Literacy Scale).

	B (SE)	Wald (*df*)	*P* value	Exponential, B (95% CI)
COHL_SUM^a^	0.22 (0.07)	9.77 (1)	<.001	1.25 (1.09-1.43)
CRHL4^b^ (reference: not really)	N/A^c^	10.41 (2)	.01	N/A
CRHL4 (yes)	0.71 (0.27)	7.00 (1)	.01	2.04 (1.20-3.46)
CRHL4 (sometimes)	0.71 (0.24)	8.83 (1)	<.001	2.04 (1.28-3.27)
Constant	−0.70 (0.45)	2.36 (1)	.13	0.50 (N/A)

^a^COHL_SUM: sum of the communicative health literacy subscale.

^b^CRHL4: item 4 of the critical health literacy subscale.

^c^N/A: not applicable.

**Table 5:** Factors and health behaviors associated with limited eHealth literacy responses (item 2 of the eHealth Literacy Scale).

	B (SE)	Wald (*df*)	*P* value	Exponential, B (95% CI)
Education (reference: postgraduate)	N/A^a^	12.42 (5)	.03	N/A
Education (year 6)	2.35 (0.67)	12.09 (1)	<.001	10.44 (2.78-39.19)
Education (year 9)	1.75 (0.64)	7.38 (1)	.01	5.75 (1.63-20.35)
Education (year 12)	1.83 (0.65)	7.97 (1)	<.001	6.23 (1.75-22.16)
Education (diploma)	1.85 (0.65)	8.18 (1)	<.001	6.39 (1.79-22.77)
Education (university)	1.88 (0.68)	7.67 (1)	.01	6.53 (1.73-24.67)
COHL3^b^ (reference: rarely)	N/A	6.83 (2)	.03	N/A
COHL3 (often)	−0.65 (0.31)	4.42 (1)	.04	0.52 (0.29-0.96)
COHL3 (sometimes)	−0.75 (0.29)	6.62 (1)	.01	0.47 (0.27-0.84)
CRHL1^c^ (reference: rarely)	N/A	8.57 (2)	.01	N/A
CRHL1 (often)	−0.84 (0.29)	8.49 (1)	<.001	0.43 (0.24-0.76)
CRHL1 (sometimes)	−0.45 (0.27)	2.72 (1)	.10	0.64 (0.37-1.09)
Constant	0.34 (0.69	0.25 (1)	.62	1.41 (N/A)

^a^N/A: not applicable.

^b^COHL3: item 3 of the communicative health literacy subscale.

^c^CRHL1: item 1 of the critical health literacy subscale.

We found that when a male participant reported “often” or “sometimes” for “making sure that they explain anything that you do not understand, when talking to a doctor or nurse,” compared with those who “rarely” did so, their odds of having trouble identifying helpful health information on the internet reduced significantly by 48% and 53%, respectively (COHL3, often: OR 0.52, 95% CI 0.29-0.96; *P*=.04; and COHL3, sometimes: OR 0.47, 95% CI 0.27-0.84; *P*=.01), as shown in Table 5.

Finally, it was found that greater interest in “finding out lots of different information about your health” (CRHL1) was associated with reduced odds of having trouble identifying helpful health information on the internet. Specifically, when a male participant reported “often” or “sometimes” for searching for diverse information about one’s own health, his odds of reporting difficulties to find useful information on the internet reduced by 57% and 36%, respectively (CRHL1, often: OR 0.43, 95% CI 0.24-0.76; *P*<.001; and CRHL1, sometimes: OR 0.64, 95% CI 0.37-1.09; *P*=.01), as shown in Table 5.

The third item (eHEAL3) was “I know what health information is available on the internet.” In our study, we coded functional health literacy as 1=often, 2=sometimes, and 3=rarely. As the 3 questions of the FHL subscale were related to one’s independence in comprehending health information (FHL1), securing others’ help when in need (FHL2), and completing official documents (FHL3), the higher the sum scores of the FHL subscale, the greater one’s functional health literacy. Our study found that with an increase of one score in the sum of FHL subscale, the odds of a male participant “not knowing what health information is available on the internet” reduced by 15% (sum of the FHL subscale: OR 0.85, 95% CI 0.74-0.97; *P*=.01), as shown in Table 6. We also found that when the level of agreement with the statement “If I take care of myself, I can avoid illness” (MHLC_A13) decreased, the odds of a male participant “not knowing what health information is available on the internet” increased; however, these changes were not statistically significant, as shown in Table 6.

The fourth item (eHEALS4) of the eHEALS was “I know how to find helpful health information the internet.” We found that education was a significant predictor of Chinese male participants’ capability to articulate strategies to find helpful web-based health information. Statistically significant increases in the odds of not having the knowledge to find helpful health information were found among Chinese male participants with year 6 education (OR 5.95, 95% CI 1.65-21.46; *P*=.01), year 9 education (OR 6.35, 95% CI 1.82-22.18; *P*<.001), 3-year college diplomas (OR 3.66, 95% CI 1.05-12.76; *P*=.04), and 4-year university degrees (OR 4.48, 95% CI 1.21-16.54; *P*=.02), as shown in Table 7.

It was interesting to find out that there were no statistically notable changes in the odds of not knowing how to find helpful web-based health information among male participants who reported either “often” or “rarely” challenging the advice from health and medical professionals based on their own research. On the contrary, when a male individual reported that he only “sometimes or maybe” challenged the advice from health professionals, his odds of not knowing how to find helpful web-based health information was reduced significantly by 51% (CRHL4, sometimes: OR 0.49, 95% CI 0.30-0.81; *P*<.001), as shown in Table 7. This finding, together with those presented in Table 4 (eHEAL1), suggests that the tendency and frequency of challenging advice from medical professionals based on one’s own research were common behaviors among Chinese male individuals lacking knowledge of “what health resources were available on the internet” (eHEALS1) and not knowing “how to find helpful health information the internet” (eHEALS4). This finding was reaffirmed by the 2 highly experienced Chinese clinicians in this study, with more than 20 years of experience at the Qilu Hospital.

**Table 6:** Factors and health behaviors associated with limited eHealth literacy responses (item 3 of the eHealth Literacy Scale).

	B (SE)	Wald (*df*)	*P* value	Exponential, B (95% CI)
FHL_SUM^a^	−0.17 (0.07)	6.10 (1)	.01	0.85 (0.74-0.97)
MHLC^b^_A13 (reference: strongly agree)	N/A^c^	12.32 (5)	.03	N/A
MHLC_A13 (strongly disagree)	0.68 (0.42)	2.61 (1)	.11	1.98 (0.86-4.54)
MHLC_A13 (moderately disagree)	0.16 (0.35)	0.21 (1)	.65	1.18 (0.59-2.35)
MHLC_A13 (slightly disagree)	0.52 (0.37)	2.00 (1)	.16	1.69 (0.82-3.50)
MHLC_A13 (slightly agree)	0.66 (0.41)	2.64 (1)	.10	1.94 (0.87-4.33)
MHLC_A13 (moderately agree)	−0.33 (0.38)	0.75 (1)	.39	0.72 (0.35-1.51)
Constant	2.02 (0.56)	13.01 (1)	<.001	7.52 (N/A)

^a^FHL_SUM: sum of the functional health literacy subscale.

^b^MHLC: Multidimensional Health Locus of Control.

^c^N/A: not applicable.

**Table 7:** Factors and health behaviors associated with limited eHealth literacy responses (item 4 of the eHealth Literacy Scale).

	B (SE)	Wald (*df*)	*P* value	Exponential, B (95% CI)
Education (reference: postgraduate)	N/A^a^	13.23 (5)	.02	N/A
Education (year 6)	1.78 (0.65)	7.41 (1)	.01	5.95 (1.65-21.46)
Education (year 9)	1.85 (0.64)	8.38 (1)	<.001	6.35 (1.82-22.18)
Education (year 12)	1.26 (0.64)	3.87 (1)	.05	3.51 (1.00-12.29)
Education (diploma)	1.30 (0.64)	4.14 (1)	.04	3.66 (1.05-12.76)
Education (university)	1.50 (0.67)	5.06 (1)	.02	4.48 (1.21-16.54)
CRHL4^b^ (reference: no)	N/A	8.02 (2)	.02	N/A
CRHL4 (often)	−0.46 (0.27)	2.81 (1)	.09	0.63 (0.37-1.08)
CRHL4 (sometimes)	−0.71 (0.25)	7.98 (1)	<.001	0.49 (0.30-0.81)
Constant	−0.24 (0.64)	0.14 (1)	.70	0.78 (N/A)

^a^N/A: not applicable.

^b^CRHL4: item 4 of the critical health literacy subscale.

The fifth item (eHEALS5) of the eHEALS was “I know how to use the health information I find on the internet to help me.” When male participants have an education level lower than the reference category (postgraduate), their odds of not knowing “how to use the health information I find on the internet to help myself” increased significantly (year 6 education: OR 6.73, 95% CI 1.62-27.99; *P*=.01; year 9 education: OR 6.60, 95% CI 1.64-26.59; *P*=.01; year 12 education: OR 11.16, 95% CI 2.71-45.93; *P*<.001; 3-year college diplomas: OR 8.48, 95% CI 2.07-34.70; *P*<.001; and 4-year university degrees: OR 6.77, 95% CI 1.60-28.54; *P*=.01), as shown in Table 8.

Additionally, when male participants reported that they were “rarely” able to “easily get hold of someone to help me when I need help” (FHL2, rarely) and simply never thought of seeking others’ help (FHL2, not applicable), their odds of not knowing “how to use the health information I find on the internet to help myself” increased significantly by 146% and 139%, respectively (FHL2, rarely: OR 2.46, 95% CI 1.31-4.65; *P*=.01; and FHL2, not applicable: OR 2.39, 95% CI 1.15-4.96; *P*=.02), as shown in Table 8.

**Table 8:** Factors and health behaviors associated with limited eHealth literacy responses (item 5 of the eHealth Literacy Scale).

	B (SE)	Wald (*df*)	*P* value	Exponential, B (95% CI)
Education (reference: postgraduate)	N/A^a^	12.57 (5)	.03	N/A
Education (year 6)	1.91 (0.73)	6.88 (1)	.01	6.73 (1.62-27.99)
Education (year 9)	1.89 (0.71)	7.04 (1)	.01	6.60 (1.64-26.59)
Education (year 12)	2.41 (0.72)	11.16 (1)	<.001	11.16 (2.71-45.93)
Education (diploma)	2.14 (0.72)	8.85 (1)	<.001	8.48 (2.07-34.70)
Education (university)	1.91 (0.73)	6.78 (1)	.01	6.77 (1.60-28.54)
FHL2^b^ (reference: often)	N/A	16.34 (3)	<.001	N/A
FHL2 (not applicable)	0.87 (0.37)	5.47 (1)	.02	2.39 (1.15-4.96)
FHL2 (rarely)	0.90 (0.32)	7.74 (1)	.01	2.46 (1.31-4.65)
FHL2 (sometimes)	−0.11 (0.25)	0.19 (1)	.66	0.90 (0.56-1.45)
Constant	−1.18 (0.72)	2.68 (1)	.10	0.31 (N/A)

^a^N/A: not applicable.

^b^FHL2: item 2 of the functional health literacy subscale.

The sixth item (eHEALS6) of the eHEALS was “I have the necessary skills to evaluate the health resources I find on the internet.” Again, the finding was similar to that presented in Table 5 regarding eHEAL2, “I know where to find helpful health information on the Internet.” Lower education predicted significant increases in the odds of male participants not “having the necessary skills to evaluate the health resources I find on the internet,” as shown in Table 9. Male participants who reported having greater interest in “finding out lots of different information about your health” (CRHL1) predicted significant decreases in the odds of self-reported lack of essential skills to evaluate web-based health resources (CRHL1, often: OR 0.45, 95% CI 0.26-0.77; *P*<.001; and CRHL1, sometimes: OR 0.58, 95% CI 0.35-0.98; *P*=.04), as shown in Table 9. In addition, higher levels of agreement with statements on the MHLC scales measuring one’s beliefs in internal drivers and strengths to stay healthy predicted significant decreases in the odds of self-reported lack of essential web-based health information appraisal skills (sum of the MHLC scales: OR 0.96, 95% CI 0.92-1.00; *P*=.03), as shown in Table 9.

The seventh item (eHEALS7) of the eHEALS was “I can distinguish between high- and low-quality health information on the internet.” The results were very similar to those related to eHEAL6 regarding health information appraisal skills: lower education levels predicted increased odds of self-reported lack of ability to ascertain the credibility and quality of web-based health information, with the largest increase in such odds identified among Chinese male participants with year 9 education (OR 10.10, 95% CI 2.53-40.24; *P*<.001), as shown in Table 10. Higher levels of belief in one’s own power to manage health predicted considerable decreases in the odds of self-reported lack of ability to ascertain the quality of web-based health information (sum of the MHLC scales: OR 0.95, 95% CI 0.92-0.99; *P*=.02).

The last item (eHEALS8) was “I feel confident in using information from the internet to make health decisions.” We found that an increase in age predicted increased odds of self-reported lack of confidence in using web-based health information to make health decisions among male Chinese participants (age: OR 1.02, 95% CI 1.01-1.04; *P*=.01), as shown in Table 11. Among participants who reported that they “often” knew to “ask the questions I need to ask when taking to a doctor or nurse,” their odds of reporting lack of self-confidence in using web-based health information to make health decisions decreased by 59% compared with those who “rarely” knew what questions to ask medical doctors (COHL2, often: OR 0.41, 95% CI 0.25-0.70; *P*<.001).

**Table 9:** Factors and health behaviors associated with limited eHealth literacy responses (item 6 of the eHealth Literacy Scale).

	B (SE)	Wald (*df*)	*P* value	Exponential, B (95% CI)
Education (reference: postgraduate)	N/A^a^	12.12 (5)	.03	N/A
Education (year 6)	1.72 (0.67)	6.53 (1)	.01	5.56 (1.49-20.75)
Education (year 9)	1.92 (0.66)	8.51 (1)	<.001	6.79 (1.87-24.59)
Education (year 12)	1.67 (0.66)	6.49 (1)	.01	5.32 (1.47-19.26)
Education (diploma)	1.41 (0.65)	4.68 (1)	.03	4.09 (1.14-14.63)
Education (university)	1.14 (0.67)	2.92 (1)	.09	3.13 (0.85-11.57)
CRHL1^b^ (reference: no)	N/A	8.44 (2)	.01	N/A
CRHL1 (often)	−0.80 (0.28)	8.36 (1)	<.001	0.45 (0.26-0.77)
CRHL1 (sometimes)	−0.54 (0.26)	4.23 (1)	.04	0.58 (0.35-0.98)
MHLC_SUM^c^	−0.04 (0.02)	4.66 (1)	.03	0.96 (0.92-1.00)
Constant	0.72 (0.79)	0.82 (1)	.36	2.05 (N/A)

^a^N/A: not applicable.

^b^CRHL1: item 1 of the critical health literacy subscale.

^c^MHLC_SUM: sum of the Multidimensional Health Locus of Control scales.

**Table 10:** Factors and health behaviors associated with limited eHealth literacy responses (item 7 of the eHealth Literacy Scale).

	B (SE)	Wald (*df*)	*P* value	Exponential, B (95% CI)
Education (reference: postgraduate)	N/A^a^	13.56 (5)	.02	N/A
Education (year 6)	1.87 (0.72)	6.82 (1)	.01	6.48 (1.59-26.32)
Education (year 9)	2.31 (0.71)	10.75 (1)	<.001	10.10 (2.53-40.24)
Education (year 12)	2.13 (0.71)	9.13 (1)	<.001	8.43 (2.11-33.63)
Education (diploma)	2.05 (0.70)	8.53 (1)	<.001	7.80 (1.97-30.96)
Education (year 6)	1.62 (0.72)	5.09 (1)	.02	5.03 (1.24-20.49)
MHLC_SUM^b^	−0.05 (0.02)	5.71 (1)	.02	0.95 (0.92-0.99)
Constant	−0.03 (0.82)	0.00 (1)	.97	0.97 (N/A)

^a^N/A: not applicable.

^b^MHLC_SUM: sum of the Multidimensional Health Locus of Control scales.

**Table 11:** Factors and health behaviors associated with limited eHealth literacy responses (item 8 of the eHealth Literacy Scale).

	B (SE)	Wald (*df*)	*P* value	Exponential, B (95% CI)
Age	0.02 (0.01)	6.27 (1)	.01	1.02 (1.01-1.04)
COHL2^a^ (reference: no)	N/A^b^	13.30 (2)	<.001	N/A
COHL2 (often)	−0.88 (0.26)	11.11 (1)	<.001	0.41 (0.25-0.70)
COHL2 (sometimes)	−0.29 (0.26)	1.23 (1)	.27	0.75 (0.45-1.25)
Constant	0.25 (0.48)	0.28 (1)	.60	1.29 (N/A)

^a^COHL2: item 2 of the communicative health literacy subscale.

^b^N/A: not applicable.

## Discussion

### Principal Findings in Relation to Previous Studies

By applying logistic regression modeling, we explored the factors associated with limited eHealth literacy among the Chinese male population. It has been found that 4 factors were significantly correlated with their limited eHealth literacy, as discussed in the following principal findings.

#### Principal Finding 1: Limited eHealth Literacy Was Strongly Associated With Lower Levels of All Aspects of Health Literacy (Functional, Communicative, and Critical)

This finding is consistent with those reported in previous studies. Jensen et al [37] found that individuals with low health literacy were less likely to access certain digital technologies, and Bailey et al [38] also discovered health literacy–related disparities in technology access and use. Similarly, as ascertained by Manganello et al [36], people with lower self-assessed health literacy were less likely to report the use of search engines to seek information on the internet. In contrast, other studies attested the association between higher eHealth literacy and higher health literacy skills. People with higher health literacy are more likely to seek health information from the internet and their health care providers [39,40]. As such, our finding that lower health literacy skills predicted lower skills essential for assessing web-based health resources aligns well with the findings of previous studies [36-40].

Interestingly, as we found, the tendency and frequency of challenging suggestions from medical professionals based on one’s own research (CRHL) were common behaviors among Chinese male individuals lacking eHealth literacy, specifically not knowing “what health resources were available on the internet” (eHEALS1) and “how to find helpful health information the internet” (eHEALS4). This finding was reaffirmed by the 2 highly experienced Chinese clinicians in this study, with more than 20 years of work at Qilu Hospital, China.

#### Principal Finding 2: Older Age Predicted Limited eHealth Literacy

This finding supports those of several previous studies. As shown by Neter and Brainin [49], older and less educated individuals have lower eHealth literacy than younger and more educated individuals [49-51]. Aaronson et al [52] reported a similar finding concerning the notable association between age and eHealth literacy [52]. This association was also identified by Knapp et al [53]; older age was correlated with lower levels of agreement on the eHEALS. This can be explained by the fact that older adults have very limited acceptance and application of digital health [54,55] because they have difficulty navigating the internet and knowing which resources to trust [56] and turning the internet health knowledge into action and applying it to health self-management [57]. The lack of eHealth literacy has become a major impediment preventing older adults from integrating into the digital society and benefiting from convenient and efficient eHealth services [57]. It is imperative to improve older adults’ perceptions of the usefulness, ease of use, and reliability of eHealth services and to reduce their perception of eHealth-related risks [58]. In this way, older individuals can be helped to accept eHealth services and change their eHealth behaviors to improve their eHealth literacy skills [58].

However, Milne et al [24] did not find a notable association between eHealth literacy and age. One possible explanation may be that “the predictive value of age on eHealth literacy may reach a ceiling effect such that after a certain age, the degree to which it affects eHealth literacy is no longer significant” [24]. Therefore, further studies need to be conducted on eHealth literacy to ascertain the predictive role of age among people living in diverse linguistic, cultural, ethnic, and socioeconomic communities.

#### Principal Finding 3: Lower Education Attainment Was an Important Predictor of Limited eHealth Literacy

Education was a notable predictor of Chinese male participants’ capability to articulate strategies to find helpful web-based health information, as found in our study. This finding confirms those of Knapp et a [53], who discovered that high eHealth literacy correlated with higher levels of educational attainment, and Nielsen-Bohlman et al [59], who revealed that people’s health literacy may be influenced by their background, such as education and situational characteristics related to health. Neter and Brainin [49], Li et al [50], and Tennant et al [51] reported similar findings, such as older, less educated people have more limited eHealth literacy skills than younger, more educated people.

#### Principal Finding 4: Limited eHealth Literacy Correlated With Weaker Beliefs and Self-confidence in Internal Drivers and Strengths to Stay Healthy

The results of our study showed that higher levels of beliefs in internal drivers and strengths to stay healthy predicted notable decreases in the odds of self-reported lack of essential web-based health information appraisal skills. This parallels the finding reported by Aponte and Nokes [60] that older adults who perceive themselves to be in good health attach great importance to health status and tend to have stronger self-care awareness and health information needs. Therefore, they are more likely to actively seek web-based health knowledge and skills and practice them in daily life, as claimed by Aponte and Nokes [60]. Thus, such individuals may have higher levels of eHealth literacy.

There is no study in the literature that has directly correlated people’s status of eHealth literacy with their “Internal” locus of control, that is, their beliefs in internal drivers and strengths to stay healthy. This study is the first to find such a direct correlation. We will conduct future studies to further ascertain this association in other Chinese populations with different demographic characteristics. Hopefully, as informed by this study, researchers will carry out similar studies to pinpoint the relationship between eHealth literacy status and the “Internal” locus of control to fill the gap in the literature.

### Implications

This study has implications for clinical practice, health education, medical research, and public health policy-making. The 4 important predictors of limited eHealth literacy could serve as important indicators for screening individuals with limited eHealth literacy skills to deliver targeted education and interventions. Knowledge, skills, beliefs, and practices related to the 4 ascertained predictive factors can be integrated into public health education on eHealth resources and interventions to improve individuals’ eHealth literacy. Medical researchers may gain insights into the topic of limited eHealth literacy and its underlying factors. Informed by this study, they can verify the factors ascertained in this study and identify additional contributors in future research. Finally, our research results and findings may provide implications for public health policy making in the future.

### Limitations

This study analyzed factors and health behaviors associated with limited eHealth literacy among Chinese male participants. However, self-reported literacy skills do not always align with the actual ability to comprehend, use, and appraise web-based health information [61]. More objective measures need to be developed to increase the reliability and consistency of digital health literacy assessments among culturally and linguistically diverse people. The second limitation concerns the generalizability of the research results and findings. The recruitment of patients from merely 1 hospital may make the results and findings less generalizable to populations in other provinces of China and to populations in different linguistic and cultural communities worldwide. Further studies are warranted to verify these factors among populations with diverse ethnic and sociocultural backgrounds.

### Conclusions

By applying logistic regression modeling, we found that limited eHealth literacy among Chinese male populations was closely associated with four factors: (1) older age, (2) lower educational attainment, (3) lower levels of all aspects of health literacy (functional, communicative, and critical), and (4) weaker beliefs and self-confidence in internal drivers and strengths to stay healthy. These predictive factors of limited male eHealth literacy can provide implications for clinical practice, health education, medical research, and health policy making.

## Appendix

Multimedia Appendix 1 Descriptive statistics of the study participants.

## Abbreviations

AAHLS: All Aspects of Health Literacy Scale
CH-eHEALS: Chinese version of the eHealth Literacy Scale
COHL: communicative health literacy
CRHL: critical health literacy
eHEALS: eHealth Literacy Scale
FHL: functional health literacy
GHNT-6: General Health Numeracy Test
MHLC: Multidimensional Health Locus of Control
OR: odds ratio
Sw-eHEALS: Swedish version of the eHealth Literacy Scale

## Data Availability

Data are available upon reasonable request to author YS (victorsyhz@hotmail.com).
