# Supplementary material for: Factors Associated With Limited Digital Health Literacy Among Chinese Male Populations: Cross-sectional Study
Source: JMIR Form Res. 2023 Apr 19;7:e42868. doi: 10.2196/42868 (PMC10157464; doi:10.2196/42868)
Supplement: Multimedia Appendix 1 [file formative_v7i1e42868_app1.docx]

**Multimedia Appendix 1. Descriptive statistics of the study participants.**

|  | Minimum | Maximum | Mean | Std. Deviation |
| --- | --- | --- | --- | --- |
| Age | 17 | 68 | 45.73 | 10.31 |
| Gender (Male) |  | | | |
| **Education^a^** | 1 | 6 | 2.91 | 1.35 |
| Disease Knowledge^b^ | 1 | 4 | 2.34 | 0.98 |
| FHL1^c^ | 1 | 3 | 2.02 | 0.77 |
| FHL2 | 1 | 4 | 2.75 | 1.00 |
| FHL3 | 1 | 3 | 2.06 | 0.75 |
| FHLSUM | 3 | 10 | 6.83 | 1.57 |
| COHL1^c^ | 1 | 3 | 1.85 | 0.77 |
| COHL2 | 1 | 3 | 1.92 | 0.75 |
| COHL3 | 1 | 3 | 1.95 | 0.75 |
| COHL_SUM | 3 | 9 | 5.73 | 1.45 |
| CRHL1^c^ | 1 | 3 | 1.99 | 0.75 |
| CRHL2 | 1 | 3 | 1.96 | 0.77 |
| CRHL3 | 1 | 3 | 1.99 | 0.77 |
| CRHL4 | 1 | 3 | 1.96 | 0.73 |
| CRHL5 | 1 | 3 | 1.94 | 0.74 |
| CRHL6 | 1 | 2 | 1.40 | 0.49 |
| eHL1^d^ | 1 | 5 | 2.73 | 1.24 |
| eHL2 | 1 | 5 | 2.69 | 1.23 |
| eHL3 | 1 | 5 | 2.71 | 1.20 |
| eHL4 | 1 | 5 | 2.89 | 1.25 |
| eHL5 | 1 | 5 | 2.66 | 1.22 |
| eHL6 | 1 | 5 | 2.76 | 1.25 |
| eHL7 | 1 | 5 | 2.73 | 1.20 |
| eHL8 | 1 | 5 | 2.85 | 1.26 |
| eHL_SUM | 13 | 40 | 22.01 | 4.50 |
| GHNT1**^e^** | 1 | 2 | 1.55 | 0.50 |
| GHNT2 | 1 | 2 | 1.12 | 0.33 |
| GHNT3 | 1 | 2 | 1.14 | 0.35 |
| GHNT4 | 1 | 5 | 1.94 | 0.27 |
| GHNT5 | 1 | 2 | 1.88 | 0.32 |
| GHNT6 | 1 | 2 | 1.84 | 0.37 |
| GHNT_SUM correct | 0 | 6 | 2.52 | 1.00 |
| Valid N (listwise) | 543 |  |  |  |

^a^ 1= Year 6, 2= Year 9, 3= Year 12, 4= diploma, 5= bachelor, 6=postgraduate

^b^ 1=very well, 2= a lot, 3= some, 4=limited

^c^ 1= often, 2=sometimes, 3=rarely

^d^ 1= strongly disagree, 2=disagree, 3=unsure, 4=agree, 5=strongly agree

^e^ 1=correct response, 2=wrong response ‘
